# Associations between a neurophysiological marker of central cholinergic activity and cognitive functions in young and older adults

**DOI:** 10.1186/1744-9081-8-17

**Published:** 2012-06-20

**Authors:** Marielle Young-Bernier, Yael Kamil, François Tremblay, Patrick SR Davidson

**Affiliations:** 1 School of Psychology, University of Ottawa, 136 Jean Jacques Lussier Private, Ottawa, Ontario, K1N 6N5, Canada; 2 Élisabeth Bruyère Research Institute, University of Ottawa, Ottawa, Ontario, Canada; 3 School of Rehabilitation Sciences, University of Ottawa, Ottawa, Ontario, Canada; 4 Heart and Stroke Foundation Centre for Stroke Recovery, University of Ottawa, Ottawa, Ontario, Canada

**Keywords:** Acetylcholine, Aging, Cortical inhibition, Executive function, Memory, Transcranial magnetic stimulation

## Abstract

**Background:**

The deterioration of the central cholinergic system in aging is hypothesized to underlie declines in several cognitive domains, including memory and executive functions. However, there is surprisingly little direct evidence regarding acetylcholine’s specific role(s) in normal human cognitive aging.

**Methods:**

We used short-latency afferent inhibition (SAI) with transcranial magnetic stimulation (TMS) as a putative marker of cholinergic activity in vivo in young (n = 24) and older adults (n = 31).

**Results:**

We found a significant age difference in SAI, concordant with other evidence of cholinergic decline in normal aging. We also found clear age differences on several of the memory and one of the executive function measures. Individual differences in SAI levels predicted memory but not executive functions.

**Conclusion:**

Individual differences in SAI levels were better predictors of memory than executive functions. We discuss cases in which the relations between SAI and cognition might be even stronger, and refer to other age-related biological changes that may interact with cholinergic activity in cognitive aging.

## Background

Normal aging is associated with declines in several cognitive domains, most notably episodic memory and executive functions (for reviews, see [1-4]). These cognitive deficits are associated with myriad brain changes, including structural and functional deterioration of prefrontal, basal ganglia, and medial temporal areas and their interconnections. However, establishing a link between these changes and cognitive decline in normal aging has proven surprisingly difficult [2,5].

Alterations in two classic neurotransmitter systems have drawn considerable attention in cognitive aging: dopamine [6] and acetylcholine. For decades, acetylcholine (ACh) was thought of primarily as a memory-related neurotransmitter, but this view has recently been revised, with ACh now thought to play an equally if not more crucial role in executive functions (for reviews, see [7-9]). The integrity of cortical cholinergic inputs appears to be critical for modulating attention, by enhancing responsiveness to sensory inputs to facilitate cue detection and orienting [10] (for a review, see [9]). Cholinergic neuromodulation may also play an important role in executive functions by selectively enhancing task-relevant inputs via bottom-up thalamic processes, while suppressing irrelevant stimuli via top-down prefrontal modulation [11] (for other perspectives, see [12,13]). This cholinergic-dependent interaction between bottom-up and top-down processes appears to be affected by aging, leading to difficulty in task-switching, handling competition among several possible responses, and suppressing unwanted responses [11]. In memory, optimal levels of ACh may facilitate encoding by increasing the influence of inputs into the hippocampus through enhanced potentiation [9,14], and/or by providing the attentional “glue” to bind together disparate elements of an episode into a unified memory trace [15,16].

Experimental and correlational animal studies, as well as computational modelling, have yielded much information on the role of the cholinergic system in cognition. However, the extent to which age-related changes in cholinergic neuromodulation contribute to cognitive decline in normal human aging remains unclear. There are at least three reasons for this: First, making inferences from animal and computational models to humans has sometimes proven surprisingly difficult (e.g., [17,18]). Second, much of what we infer about the role of ACh in cognitive aging comes from studies in which Alzheimer’s patients are treated with cholinesterase inhibitors, including donepezil, galantamine, and rivastigmine (e.g., [19]). Unfortunately, these patients can be difficult to test and experience other confounding factors including significant structural and functional brain changes. Third, manipulation of ACh via agonist and antagonist drugs (e.g., scopolamine) has produced a vast amount of data, but strictly speaking this line of research tells us more about acute effects than it does about the long term decline in cholinergic activity seen in normal aging. There is thus a need to further examine the in vivo contribution of age-related alterations in central cholinergic function to declines in human cognition.

Recent advances in the field of non invasive brain stimulation have yielded new opportunities to examine the neurophysiological correlates of aging using markers of cortical excitability that can be linked with relative confidence to specific neurotransmitter systems [20]. One such marker involves pairing afferent nerve stimulation with transcranial magnetic stimulation (TMS) of the motor cortex to modulate motor responses evoked in contralateral hand muscles [21]. When applied at short intervals (e.g., 18–20 milliseconds [ms]) before TMS pulses, afferent nerve stimulation typically leads to a period of inhibition of the motor evoked potentials (MEPs). This *short-interval afferent inhibition* (SAI) is mediated at the cortical level through cholinergic-dependent GABA_A_ receptor activation [22]. The implication of cholinergic action in mediating SAI is supported by in vivo observations of its reduction or even abolition by administration of a selective muscarinic cholinergic receptor blocker (scopolamine) in healthy participants [23]. Further, SAI is lower than expected in Alzheimer’s patients but restored by cholinesterase inhibitors [22]. SAI is also reduced in other disorders characterized by cholinergic dysfunction, including Lewy body dementia [24], multiple sclerosis [25], and Wernicke–Korsakoff syndrome [26], but it is normal in frontotemporal dementia, a non-cholinergically mediated form of dementia [27]. Together, these observations provide strong evidence that SAI is a cholinergic-dependent marker of motor intra-cortical excitability.

Given the clear decline in cholinergic modulation with age [28,29], one would predict that SAI would be altered in healthy older adults. Yet, very few studies have examined this issue. Oliviero et al. [30] compared SAI levels in healthy young and older adults and found no age differences. More recently, Degardin et al. [31] performed a similar study and reached a similar conclusion. However, as we and others [32] have argued previously, the use of varying test intensities to obtain a constant MEP size across participants might have contributed to masking any age effects in the two studies above. In line with this, we recently found a large and selective decrease in SAI in healthy seniors when we used a constant TMS test intensity approach [33]. Further, we found that age-related variations in SAI explained a substantial proportion of the variance in timed motor tasks assessing processing speed.

This study constitutes an extension of our previous findings; data were derived from the same sample of participants as already described [33]. In the present study, we examined possible relationships between SAI, as a putative marker of cholinergic-dependent cortical inhibition, and cognition in young and older healthy adults. Because mean differences between young and older adult groups are often small, especially relative to the extensive variability that can be seen among healthy older adults (e.g., some perform much more poorly than young people, whereas others are indistinguishable from the young [34]), we capitalized on the individual-differences approach used by Glisky and colleagues [35,36]. This approach allows the characterization of each participant’s long-term memory and executive functions using neuropsychological testing to construct aggregate scores reflecting performance across several tasks in each domain (for details, see Method). We hypothesized that age-related differences in SAI levels would be associated with age-related differences in memory and executive functions. For memory, several investigators have emphasized ACh’s putative role in binding information in memory [15], which we assessed using a canonical measure of paired associate learning (Verbal Paired Associates from the Wechsler Memory Scale-III; WMS-III [37]). We also examined face recognition from the WMS-III because recent studies have also described cholinergic modulation of face-memory-related activity in the fusiform gyrus [38]. Given the emphasis in the recent literature on the crucial role of ACh in modulating executive functions [19,39,40], we also expected correlations between SAI and our aggregate executive function measure.

## Method

### Participants

The present data were derived from the same group of participants previously described [33], with minor differences in the current sample (i.e. one young adult was excluded from the present study because of incomplete cognitive data). We analyzed data from 24 young adults (age range = 18 to 30 years; M = 22.67, SD = 3.49; 13 females) and 31 community-dwelling older adults (age range = 65 to 82 years; M = 70.29, SD = 3.81; 18 females). The two age groups were similar in education (young: M = 16.08 years, SD = 1.89; older adults: M = 16.19, SD = 2.83). All participants were fluent English and/or French speakers with normal or corrected-to-normal vision (one participant was blind in one eye, but had no difficulty with the visual tasks) and hearing, and were screened for depression (two participants were taking anti-depressants but their depression screening scores, TMS, and cognitive data were normal), dementia, psychiatric or neurological disorders, drug or alcohol abuse, and counter-indications to TMS. Participants’ medications were not altered for testing, with many older adults taking drugs related to vascular health (e.g., hypertension, statins cholesterol lowering drugs). None of the participants was taking neuroactive drugs such as neuroleptics, however one young adult and one older adult were taking antidepressants (as mentioned above, their TMS data were normal). Vascular risk factors were assessed for each participant and consisted of a cumulative score of 6 factors: body mass index with obesity defined as being greater than 30 kg/m^2^, current smoking status, lack of physical activity, type-2 diabetes, history of hypertension, and history of cardiac symptoms [41,42]. Vascular risk factors for participants ranged from 0 to 3 (M = 0.44) with the maximum possible score being 6, suggesting generally good vascular health. All participants also completed the Montreal Cognitive Assessment (MoCA; [43]). Although some older adults (5/31) scored slightly below the recommended cutoff (i.e., >26), they were deemed eligible for the study based on the interview and their good performance on the other tasks, and on recent evidence that this cut-off may be too high [44]. The results of five additional participants were discarded because they did not meet inclusion criteria and thirteen more (including 6 older adults) because of incomplete testing (10 could not be reached for a second testing session resulting in missing TMS-SAI data and 3 decided to stop before completion). The Research Ethics Boards of the University of Ottawa and Bruyère Continuing Care approved the study procedure in accordance with the principles of the Declaration of Helsinki. Informed consent was obtained from each participant before the experimental session and all volunteers received a minimal honorarium to defray expenses for participation.

### TMS procedure for short-afferent inhibition

The TMS procedure has been reported in detail previously [33]. In brief, motor evoked potentials (MEP) were recorded using small pairs of auto-adhesive surface electrodes (10 mm diameter, Ag-AgCl) placed over the first dorsal interosseous (FDI) muscle of the right hand. Electromyographic signals were amplified (100–500 mV/div) and filtered (bandwidth, 10 Hz to 1 kHz) with a polygraph amplifier (RMP-6004, Nihon-Kohden Corp.; BNC-2090, National Instrument Corp.). Magnetic stimulation was delivered with a Magstim Rapid^2^ stimulator (Magstim Co. Dyfed, UK) connected to a figure-eight coil (90-mm inside loop diameter), held ~45° in the mid-sagittal plane. The resting motor threshold (RMT) was determined using the method of Mills and Nithi [45]: the RMT was defined for each participant as the median intensity between the upper and lower threshold values. The test TMS intensity was fixed at 120% RMT for both unconditioned and conditioned trials. Conditioning afferent stimulation was produced by applying 200 μs electrical pulses (S88 Stimulator, Grass Technologies, Astro-Med, Inc, West Warwick, RI 02893 U.S.A.) on the median nerve at an intensity just above the motor threshold to evoke a minimal visible twitch of the thenar muscles [23,46]. SAI was measured by applying afferent stimulation 20 ms before the TMS pulse over the motor cortex. Other inter-stimulus intervals (ISI; 25, 50 or 200 ms; see [33]) were also investigated. Unconditioned MEP amplitude was first determined for each participant by eliciting 15 MEPs at rest (120% RMT). Following the same procedure, blocks of trials were made for each conditioned interval (order was counterbalanced across participants). Trials for which unwanted contractions were present were eliminated and repeated if necessary.

### Analysis of MEP data

Mean individual values for conditioned and unconditioned MEP responses were measured off-line by averaging the amplitude (peak-to-peak) and latency of each trial. SAI level was determined in each participant in terms of percent of unconditioned MEP responses (i.e.% MEP _Conditioned_/MEP_Unconditioned_).

### Memory and executive functions

Participants underwent neuropsychological testing in a quiet, well-lit room, in their language of choice. We created two composite *z* scores for each individual, based on previous factor analyses [35,36]. The first factor score reflects long-term memory and is composed of five scores: the Logical Memory I, Faces recognition I, and Verbal Paired Associates I subtests of the WMS-III, Visual Paired Associates II from the Wechsler Memory Scale–Revised (WMS-R; [47]), and Long Delay Cued Recall from the California Verbal Learning Test-II (CVLT-II; [48]). The second factor score, reflecting executive function, is made up of the number of categories achieved on the computerized Wisconsin Card Sorting Test [49], the total number of words produced to the cues *FA*, and *S* on a phonemic fluency test [50], and the Backward Digit Span and Mental Control measures from the WMS–III. In previous studies involving only older adults, the executive function factor had also included Mental Arithmetic from the Wechsler Adult Intelligence Scale—Revised (WAIS-R; [51]), but [35] reported that this measure did not load significantly on the executive function factor in their young adults. Therefore, we omitted this measure from the executive function *z* score in both groups to allow for direct age group comparisons.

### Statistical methods

Independent *t*-tests, with adjusted *p* values for multiple comparisons (i.e. *p* = 0.0125), were used to examine age group differences on baseline measures of excitability. Mixed analysis of variance (ANOVA) and independent *t*-tests were used to examine differences between age groups. We adjusted *p* values to correct for multiple comparisons in the between-group *t*-tests on the cognitive tasks (*p* = 0.05/8, that is, *p* = 0.00625). We used Pearson’s correlations to examine associations among SAI levels and memory and executive function scores. All statistical tests were performed using the PASW software version 18.0 for Windows® (Chicago, IL, USA). The figure was prepared with GraphPad Prism version 5.00 for Windows (GraphPad Software, San Diego California USA, http://www.graphpad.com).

## Results

### TMS and SAI

The TMS procedure was well tolerated and no participants experienced adverse effects. A thorough analysis of the physiological data has been reported previously [33] (see Table 1 for baseline TMS measurements). Briefly, young adults generally exhibited marked MEP suppression in response to afferent conditioning leading to high levels of SAI (18.13 ± 15.74). In contrast, seniors exhibited more variable afferent-induced inhibition with a substantial proportion of subjects (14/31) showing either low or absent inhibition (MEP_cond_ ≥ 50% suppression). Accordingly, SAI levels estimated in seniors (51.36 ± 34.62) were significantly lower than in young adults (*p* < 0.001). .

**Table 1:** Hand dominance and baseline measures of excitability in the two age groups (mean ± SD)

	Young (n = 24)	Senior (n = 31)
Hand Dominance (L/R)	2/22	1/30
Resting MT (% output)	66.00 ± 11.55	72.55 ± 12.71
Test MT (% output)	79.17 ± 13.82	86.97 ± 15.15
Resting MEP amplitude (μV)	926.61 ± 774.34	427.22 ± 540.59*
Resting MEP latency (ms)	22.27 ± 1.88	24.03 ± 1.87*
Intensity MNS^1^	64.17 ± 1.80	72.87 ± 1.72

Key: MEP, motor evoked potential; MNS, median nerve stimulation; MT, motor threshold.

^1^ Conditioning intensity for median nerve stimulation (MNS).

*Significant difference at adjusted *p*-values (p = 0.0125) for multiple comparisons (see [33] for a more elaborate analysis of these age differences)

### Age differences in cognition

The young adults performed significantly better on several of the memory and executive function tasks than the older adults did (ANOVA: main effect of Age: *F*_1,51_ = 6.86, *p* = 0.01, significant Age X Task interaction: *F*_7, 357_ = 3.22; *p* = 0.003^a^). At the adjusted *p* value, post-hoc *t* tests showed that the young significantly outperformed the older adults on memory for Verbal Paired Associates I (*t*_53_ = 4.03, *p* = 0.0002) and Faces I (*t*_52_ = 3.89, *p* = 0.0003), and number of categories on the Wisconsin Card Sorting Test (*t*_52_ = 4.10, *p* = 0.0001). Although the two age groups could not be compared on the Visual Paired Associates II measure using parametric methods because of ceiling effects in the young adults (that is, all the young adults scored 6 out of 6, whereas the older adults ranged from 4 to 6), a Chi-Squared analysis suggested a significant advantage for the young adults (χ_2_^2^ = 9.82, *p* = 0.007). The factor scores, by definition, reflected the individual test scores: The young had significantly higher scores than the older adults on the memory factor *z* score (*t*_53_ = 4.53, *p* < 0.0001), but the groups were not significantly different from one another on the executive function factor *z* score (*t*_53_ = 1.65, *p* = 0.11). The mean levels of performance on the individual cognitive tasks and the factor scores are shown in Table 2.

**Table 2:** Cognitive performance in the two age groups (mean ± SD)

	Young Adults (n = 24)	Older Adults (n = 31)
Logical Memory I	30.46 ± 4.04	29.00 ± 6.77
Visual Paired Associates II	6.00 ± 0.00	5.50 ± 0.77 ***
Verbal Paired Associates I	26.63 ± 5.59	19.00 ± 7.84 ***
Faces I	38.71 ± 4.31	34.67 ± 3.34 ***
CVLT-II Long-Delay Cued Recall^1^	13.67 ± 1.81	12.39 ± 2.70
Verbal Fluency (FAS) Test	40.25 ± 9.81	41.00 ± 12.20
Backward Digit Span	7.67 ± 2.67	7.42 ± 2.80
Wisconsin Card Sorting Test	4.25 ± 0.85	2.83 ± 1.51 ***
Mental Control	27.13 ± 4.74	26.39 ± 4.10
Memory factor (*z* score)	0.39 ± 0.39	−0.31 ± 0.68 ***
Executive function factor (*z* score)	0.16 ± 0.50	−0.12 ± 0.71

^1^ California Verbal Learning Test-II.

Significant difference at adjusted p-values (p = 0.006) for multiple comparisons

*** *p* < 0.001.

### Correlations between SAI and cognition

**Figure 1:**
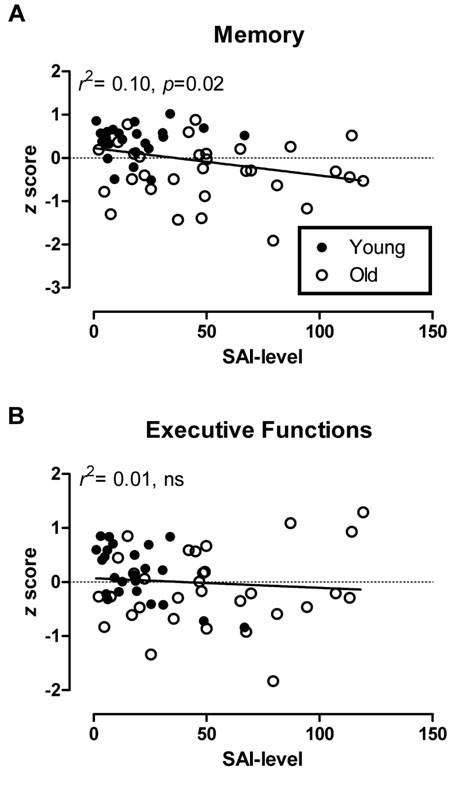
**Scatter plots showing the associations between SAI levels and composite*z*scores of (A) memory and (B) executive functions.** SAI levels correspond to the modulation of motor evoked potentials (MEP) induced by afferent conditioning at an inter-stimulus interval (ISI) of 20 ms (% Conditioned MEP/Unconditioned MEP).

When we performed an analysis across all individuals [52,53]; but see [54,55], SAI significantly predicted the memory factor score (*r* = −0.31, *p* = 0.02), whereas it did not predict the executive function *z* score (*r* = −0.09, *p* = 0.51; see Figure 1). The correlation between SAI and memory was modest in size (*r*^2^ = 10%), and when we examined the correlation separately within each age group it failed to obtain significance. Although in the young group alone a significant correlation between SAI levels and the executive function *z* score emerged in our initial analysis (*r* = −0.56, *p* = 0.004), visual inspection suggested that this was driven by two data points; indeed, if we deleted these two cases the correlation was rendered non-significant.

Based on the hypotheses outlined at the end of the introduction, we also examined associations between SAI and specific individual subtest scores. First, we found a significant correlation between SAI and Verbal Paired Associates I (*r* = −0.35, *p* = 0.008), a canonical measure of memory binding, although this correlation became non-significant when we examined each age group on its own (*r ≤* |0.21|). Note that although Visual Paired Associates II is also a canonical measure of this ability, it was not explored further because of the ceiling-level scores in data, especially for the young adults. Second, we found a significant correlation between SAI and memory for faces (Faces I; *r* = −0.31, *p* = 0.02), although, again, it disappeared when analyses were performed separately within each age group (*r ≤* |0.17|).

We also performed all analyses while excluding the five older adults who had MoCA scores lower than the recommended cutoff. This did not yield any changes in the results.

## Discussion

Deficits in central cholinergic activity are thought to underlie age-related cognitive decline, but evidence regarding the specific role(s) of ACh in human cognitive aging is still scarce. We investigated the relation of SAI, a putative neurophysiological marker of cholinergic activity, to memory and executive functions in aging.

### Age differences in SAI

Consistent with reports of impaired cortical inhibition with age [56], as a group, our senior participants exhibited reduced intra-cortical inhibition, as reflected in the overall decrease in afferent-induced inhibition. The fact that SAI has been linked with cholinergic activity in the motor cortex in pharmacological and patient studies (e.g., [22,23,57]; but see below) provides further converging in vivo evidence of a decline in central cholinergic function in normal human aging (e.g., [58]for reviews, see [28,59]).

### Associations between SAI and cognition

The young adults outperformed their older counterparts on several measures of memory, consistent with numerous previous reports [1-4]. Although memory was clearly impaired in the older adults, executive function was not. This finding is concordant with a similar study to ours [35], which noted that others too have found this pattern. For example, Lamar and Resnick [60] reported no age differences in verbal fluency, mental control, and digit span, which were included in the present executive function factor score.

SAI predicted individual performance in memory, although, contrary to expectations, it did not predict executive functioning. These results are consistent with some studies [61], but not with others [19,39,40] and may stem from the poor vascular health of the patients included in those studies. (This issue will be discussed further below.) The association between SAI and memory is also consistent with Duzel et al. [52], who recently reported that a magnetic resonance imaging estimate of the structural integrity of the basal forebrain (the major source of cholinergic input into the cortex and hippocampus) predicted verbal memory in a mixed sample of young and older adults.

In the present study, SAI levels explained approximately 10% of the variance in memory. Although this is comparable in size to the explanatory power of Duzel et al.’s [52] measure of basal forebrain integrity, we suspect that the relation between SAI and cognition might be even stronger under different circumstances. First, pharmacological studies indicate that ACh must decline past a certain threshold before changes in cognition are detectable [62-66]. Although we studied a representative group of older adults, only a small number of them exhibited relatively low SAI levels. Given that cholinergic function declines with age, one future possibility would be to recruit older seniors (i.e., over 80 years of age) with the expectation that stronger correlations with cognition would emerge. Also, one important putative cause of cholinergic decline in aging is microvascular damage to the ascending cholinergic pathways from the midbrain to the cortex [67,68]. Our older participants were in relatively good vascular health. Were we to focus on recruiting people in poorer vascular health, we might find stronger correlations between cholinergic function and cognition [39,40].

Second, it is possible that cholinergic modulation supports only relatively specific aspects of memory and executive functions and that these processes were not optimally assayed or taxed by the current neuropsychological battery. A general assertion is that for ACh to be significantly implicated in cognitive tasks, these tasks must be difficult and require effortful attention [11,59]. The tasks in the current study all fit this description. However, based on techniques that can target specifically the cholinergic system in animals (e.g., the immunotoxin 192 IgG-saporin), it has recently been argued that ACh is particularly important for certain memory functions, including encoding more so than retrieval, and remembering relational and contextual information in particular [15,69]. Consistent with the strong involvement of Ach in attention, studies have also suggested that the cholinergic system is more important for strategic and effortful processing of information to be remembered rather than when it is automatic [70]. Regarding executive functions, cholinergic activity may be especially important for task-switching, handling competition among possible responses, and suppressing unwanted responses [11]. Although we did measure several of these putative processes (e.g., memory binding with the visual and verbal paired associates subtests; switching and suppression with the Wisconsin Card Sorting Test), we are currently developing a new battery to probe some of these memory and executive sub-processes more specifically. Combined with our previous observation of an association between SAI and complex motor tasks (i.e. Grooved Pegboard Test, complex reaction times, go/no-go) but not with simple reaction times in aging [33], this study suggests that SAI may be a better predictor of memory than executive functions, but an even stronger indicator of motor performance and information processing speed.

Third, recent microdialysis studies have described phasic cholinergic release during attention-related tasks in rats [71,72]. These studies suggest that indices of relatively tonic ACh levels (including SAI, positron emission tomography, and magnetic resonance spectroscopy) in the brain will need to be supplemented with methods that have higher temporal resolution when they become available in humans. Finally, like most studies, this one was cross-sectional. Complementary longitudinal studies of within-subject changes must be completed to yield a more complete understanding of the relationship between the onset and course of cholinergic dysfunction and cognitive decline in normal and pathological aging (e.g., [73]cf. [74,75]).

Strong evidence that SAI is a reliable marker of cholinergic function comes from pharmacological and patient studies [22,23,57], but gamma-aminobutyric acid (GABA), dopamine, and serotonin may also contribute to the signal (e.g., [76,77]). For example, as we have noted previously [33], our older adults showed greater inter-individual variability in SAI than did our young adults, with approximately half the seniors exhibiting either poor or absent intra-cortical inhibition. These older adults were indistinguishable from the other seniors in terms of age and vascular health, and there was no evidence that these individuals were in a preclinical stage of dementia. One possibility, however, is that these individual differences in intra-cortical inhibition are related to variability in changes in motor cortex GABA_A_ receptors in aging [78,79]. Future pharmacological and neuroimaging work must verify that SAI is strongly, although perhaps not exclusively, reflective of activity in the cholinergic system.

## Conclusion

We found that individual differences in episodic memory could be explained in part by SAI, a putative marker of central cholinergic functioning. However, cholinergic decline is only one of many brain changes that occur in aging [80-82]. The goal of future research on the biological bases of cognitive aging should be to combine multiple methods to increase explanatory power, for example by combining multiple neuroimaging methods (e.g., [83,84]) with genetic information (e.g., [52,85]). The short afferent inhibition marker of cholinergic integrity reported in this study is a minimally-invasive, relatively inexpensive, significant predictor of cognition. Combining it with neuroimaging, genetic, and other cognitive neuroscience methods should prove useful in future studies.

## Endnote

^a^Three older adults each did not complete one cognitive measure (Faces I, Wisconsin Card Sorting Test and Visual Paired Associates II); their factor *z* scores were calculated by computing the mean of the remaining tests.

## Abbreviations

Ach = Acetylcholine; GABA = Gamma-aminobutyric acid; CVLT-II = California Verbal Learning Test - II; ISI = Inter-stimulus interval; MEP = Motor evoked potentials; MoCA = Montreal Cognitive Assessment; RMT = Resting motor threshold; SAI = Short-latency afferent inhibition; TMS = Transcranial magnetic stimulation; WAIS-R = Wechsler Adult Intelligence Scale – Revised; WMS-III = Wechsler Memory Scale – III; WMS-R = Wechsler Memory Scale – Revised.

## Competing interests

We declare no actual or potential conflicts of interest.

## Authors’ contributions

MYB participated in the design of the study, carried out the cognitive and behavioural testing, performed the statistical analyses, and drafted the manuscript. YK participated in the cognitive testing. FT conceived of the study, participated in its design, and helped with the behavioural testing. PD conceived of the study, participated in its design, helped with the statistical analyses and drafted the manuscript. All authors read and approved the final manuscript.

## References

[B1] Davidson PSR Winocur G Koob GF, Moal M, Thompson RF Aging and Cognition Encyclopedia of Behavioral Neuroscience, Volume 1 2010 Academic Press, Oxford 20 26

[B2] Park DC Reuter-Lorenz P The adaptive brain: aging and neurocognitive scaffolding Annu Rev Psychol 2009 60 173 196 10.1146/annurev.psych.59.103006.093656 19035823 PMC3359129

[B3] Drag LL Bieliauskas LA Contemporary review 2009: cognitive aging J Geriatr Psychiatry Neurol 2010 23 75 93 10.1177/0891988709358590 20101069

[B4] Glisky EL Riddle DR Changes in cognitive function in human aging Brain aging: Models, methods and mechanisms 2007 2011/01/05 CRC Press, Boca Raton, FL 4 20

[B5] Salthouse TA Neuroanatomical substrates of age-related cognitive decline Psychol Bull 2011 137 753 784 21463028 10.1037/a0023262 PMC3132227

[B6] Bäckman L Lindenberger U Li S-C Nyberg L Linking cognitive aging to alterations in dopamine neurotransmitter functioning: recent data and future avenues Neurosci Biobehav Rev 2010 34 670 677 10.1016/j.neubiorev.2009.12.008 20026186

[B7] Floresco SB Jentsch JD Pharmacological enhancement of memory and executive functioning in laboratory animals Neuropsychopharmacol 2011 36 227 250 10.1038/npp.2010.158 PMC3055518 20844477

[B8] Picciotto MR Meenakshi A Jentsch JD Davis KL, Charney D, Coyle JT, Nemeroff C Acetylcholine Neuropsychopharmacology: The Fifth Generation of Progress 2002 Lippincott Williams & Wilkins, Philadelphia, PA 3 14

[B9] Hasselmo ME Sarter M Modes and models of forebrain cholinergic neuromodulation of cognition Neuropsychopharmacol 2011 36 52 73 10.1038/npp.2010.104 PMC2992803 20668433

[B10] Davidson MC Marrocco RT Local infusion of scopolamine into intraparietal cortex slows covert orienting in rhesus monkeys J Neurophysiol 2000 83 1536 1549 10712478 10.1152/jn.2000.83.3.1536

[B11] Sarter M Hasselmo ME Bruno JP Givens B Unraveling the attentional functions of cortical cholinergic inputs: interactions between signal-driven and cognitive modulation of signal detection Brain Res Rev 2005 48 98 111 10.1016/j.brainresrev.2004.08.006 15708630

[B12] Yu AJ Dayan P Acetylcholine in cortical inference Neural Netw 2002 15 719 730 10.1016/S0893-6080(02)00058-8 12371522

[B13] Yu AJ Dayan P Uncertainty, neuromodulation, and attention Neuron 2005 46 681 692 10.1016/j.neuron.2005.04.026 15944135

[B14] Hasselmo ME Giocomo LM Cholinergic modulation of cortical function J Mol Neurosci 2006 30 133 135 10.1385/JMN:30:1:133 17192659

[B15] Botly LC De Rosa E A cross-species investigation of acetylcholine, attention, and feature binding Psychol Sci 2008 19 1185 1193 10.1111/j.1467-9280.2008.02221.x 19076492

[B16] Botly LC De Rosa E Cholinergic influences on feature binding Behav Neurosci 2007 121 264 276 17469916 10.1037/0735-7044.121.2.264

[B17] Arnsten AF Robbins TW Stuss DT, Knight RT Neurochemical modulation of prefrontal cortical function in humans and animals Principles of Frontal Lobe Function 2002 Oxford University Press, New York, NY 51 84

[B18] Graef S Schonknecht P Sabri O Hegerl U Cholinergic receptor subtypes and their role in cognition, emotion, and vigilance control: an overview of preclinical and clinical findings Psychopharmacol 2011 215 205 229 10.1007/s00213-010-2153-8 21212938

[B19] Behl P Lanctot KL Streiner DL Guimont I Black SE Cholinesterase inhibitors slow decline in executive functions, rather than memory, in Alzheimer’s disease: a 1-year observational study in the Sunnybrook dementia cohort Curr Alzheimer Res 2006 3 147 156 10.2174/156720506776383031 16611015

[B20] Reis HJ Guatimosim C Paquet M Santos M Ribeiro FM Kummer A Schenatto G Salgado JV Vieira LB Teixeira AL Palotas A Neuro-transmitters in the central nervous system & their implication in learning and memory processes Curr Med Chem 2009 16 796 840 10.2174/092986709787549271 19275596

[B21] Chen R Cros D Curra A Di Lazzaro V Lefaucheur JP Magistris MR Mills K Rosler KM Triggs WJ Ugawa Y Ziemann U The clinical diagnostic utility of transcranial magnetic stimulation: report of an IFCN committee Clin Neurophysiol 2008 119 504 532 10.1016/j.clinph.2007.10.014 18063409

[B22] Di Lazzaro V Oliviero A Tonali PA Marra C Daniele A Profice P Saturno E Pilato F Masullo C Rothwell JC Noninvasive in vivo assessment of cholinergic cortical circuits in AD using transcranial magnetic stimulation Neurology 2002 59 392 397 10.1212/WNL.59.3.392 12177373

[B23] Di Lazzaro V Oliviero A Profice P Pennisi MA Di Giovanni S Zito G Tonali P Rothwell JC Muscarinic receptor blockade has differential effects on the excitability of intracortical circuits in the human motor cortex Exp Brain Res 2000 135 455 461 10.1007/s002210000543 11156309

[B24] Di Lazzaro V Pilato F Dileone M Saturno E Profice P Marra C Daniele A Ranieri F Quaranta D Gainotti G Tonali PA Functional evaluation of cerebral cortex in dementia with Lewy bodies NeuroImage 2007 37 422 429 10.1016/j.neuroimage.2007.05.003 17570682

[B25] Cucurachi L Immovilli P Granella F Pavesi G Cattaneo L Short-latency afferent inhibition predicts verbal memory performance in patients with multiple sclerosis J Neurol 2008 255 1949 1956 10.1007/s00415-008-0041-5 18854915

[B26] Nardone R Bergmann J De Blasi P Kronbichler M Kraus J Caleri F Tezzon F Ladurner G Golaszewski S Cholinergic dysfunction and amnesia in patients with Wernicke-Korsakoff syndrome: a transcranial magnetic stimulation study J Neural Transm 2010 117 385 391 10.1007/s00702-009-0347-1 19960210

[B27] Di Lazzaro V Pilato F Dileone M Saturno E Oliviero A Marra C Daniele A Ranieri F Gainotti G Tonali PA In vivo cholinergic circuit evaluation in frontotemporal and Alzheimer dementias Neurology 2006 66 1111 1113 10.1212/01.wnl.0000204183.26231.23 16606932

[B28] Bartus RT On neurodegenerative diseases, models, and treatment strategies: lessons learned and lessons forgotten a generation following the cholinergic hypothesis Exp Neurol 2000 163 495 529 10.1006/exnr.2000.7397 10833325

[B29] Gallagher M Colombo PJ Ageing: The cholinergic hypothesis of cognitive decline Curr Opin Neurobiol 1995 5 161 168 10.1016/0959-4388(95)80022-0 7620303

[B30] Oliviero A Profice P Tonali PA Pilato F Saturno E Dileone M Ranieri F Di Lazzaro V Effects of aging on motor cortex excitability Neurosci Res 2006 55 74 77 10.1016/j.neures.2006.02.002 16584795

[B31] Degardin A Devos D Cassim F Bourriez JL Defebvre L Derambure P Devanne H Deficit of sensorimotor integration in normal aging Neurosci Lett 2011 498 208 212 10.1016/j.neulet.2011.05.010 21600958

[B32] Garry MI Thomson RH The effect of test TMS intensity on short-interval intracortical inhibition in different excitability states Exp Brain Res 2009 193 267 274 10.1007/s00221-008-1620-5 18974984

[B33] Young-Bernier M Davidson PS Tremblay F Paired-pulse afferent modulation of TMS responses reveals a selective decrease in short latency afferent inhibition with age Neurobiol Aging 2012 835 e1-835 e11 21958964 10.1016/j.neurobiolaging.2011.08.012

[B34] Gunstad J Paul RH Brickman AM Cohen RA Arns M Roe D Lawrence JJ Gordon E Patterns of cognitive performance in middle-aged and older adults: a cluster analytic examination J Geriatr Psychiatry Neurol 2006 19 59 64 10.1177/0891988705284738 16690989

[B35] Glisky EL Kong LL Do young and older adults rely on different processes in source memory tasks? A neuropsychological study J Exp Psychol Learn 2008 34 809 822 10.1037/0278-7393.34.4.809 PMC2504728 18605870

[B36] Glisky EL Rubin SR Davidson PS Source memory in older adults: an encoding or retrieval problem? J Exp Psychol Learn 2001 27 1131 1146 10.1037//0278-7393.27.5.1131 11550742

[B37] Wechsler D Wechsler Memory Scale-III 1997 Psychological Corporation, San Antonio, TX

[B38] Sperling R Greve D Dale A Killiany R Holmes J Rosas HD Cocchiarella A Firth P Rosen B Lake S Functional MRI detection of pharmacologically induced memory impairment Proc Natl Acad Sci U S A 2002 99 455 460 10.1073/pnas.012467899 11756667 PMC117581

[B39] Behl P Bocti C Swartz RH Gao F Sahlas DJ Lanctot KL Streiner DL Black SE Strategic subcortical hyperintensities in cholinergic pathways and executive function decline in treated Alzheimer patients Arch Neurol 2007 64 266 272 10.1001/archneur.64.2.266 17296844

[B40] Swartz RH Sahlas DJ Black SE Strategic involvement of cholinergic pathways and executive dysfunction: does location of white matter signal hyperintensities matter? J Stroke Cerebrovasc Dis 2003 12 29 36 10.1053/jscd.2003.5 17903901

[B41] Wiederkehr S Laurin D Simard M Verreault R Lindsay J Vascular risk factors and cognitive functions in nondemented elderly individuals J Geriatr Psychiatry Neurol 2009 22 196 206 10.1177/0891988709335797 19487580

[B42] Kuczynski B Jagust W Chui HC Reed B An inverse association of cardiovascular risk and frontal lobe glucose metabolism Neurology 2009 72 738 743 10.1212/01.wnl.0000343005.35498.e5 19237703 PMC2677543

[B43] Nasreddine ZS Phillips NA Bedirian V Charbonneau S Whitehead V Collin I Cummings JL Chertkow H The Montreal Cognitive Assessment, MoCA: a brief screening tool for mild cognitive impairment J Am Geriatr Soc 2005 53 695 699 10.1111/j.1532-5415.2005.53221.x 15817019

[B44] Rossetti HC Lacritz LH Cullum CM Weiner MF Normative data for the Montreal Cognitive Assessment (MoCA) in a population-based sample Neurology 2011 77 1272 1275 10.1212/WNL.0b013e318230208a 21917776

[B45] Mills KR Nithi KA Corticomotor threshold is reduced in early sporadic amyotrophic lateral sclerosis Muscle Nerve 1997 20 1137 1141 10.1002/(SICI)1097-4598(199709)20:9<1137::AID-MUS7>3.0.CO;2-9 9270669

[B46] Tokimura H Di Lazzaro V Tokimura Y Oliviero A Profice P Insola A Mazzone P Tonali P Rothwell JC Short latency inhibition of human hand motor cortex by somatosensory input from the hand J Physiol 2000 523 Pt 2 503 513 10699092 10.1111/j.1469-7793.2000.t01-1-00503.x PMC2269813

[B47] Wechsler D Wechsler Memory Scale-Revised 1987 Psychological Corporation, New York

[B48] Delis DC Kramer J Kaplan E Ober BA The California Verbal Learning Test 2000 2 Psychological Corporation, San Antonio, TX

[B49] Kongs S Thompson LL Iverson GL Heaton RK Wisconsin Card Sorting Test-64 Card Version 2000 Psychological Assessment Resources, Lutz, FL

[B50] Spreen O Benton AL Neurosensory Center Comprehensive Examination for Aphasia, Revised edition 1977 University of Victoria Neuropsychology Laboratory, Victoria, BC

[B51] Wechsler D Weschler Adult Intelligence Scale-Revised 1981 Psychological Corporation, New York

[B52] Duzel S Munte TF Lindenberger U Bunzeck N Schutze H Heinze HJ Duzel E Basal forebrain integrity and cognitive memory profile in healthy aging Brain Res 2010 1308 124 136 19857471 10.1016/j.brainres.2009.10.048

[B53] Clark J Loftus A Hammond G Age-related changes in short-interval intracortical facilitation and dexterity NeuroReport 2011 22 499 503 10.1097/WNR.0b013e3283487480 21666519

[B54] Baxter MG Gallagher M Neurobiological substrates of behavioral decline: models and data analytic strategies for individual differences in aging Neurobiol Aging 1996 17 491 495 10.1016/0197-4580(96)00011-5 8725914

[B55] Lazic SE The problem of pseudoreplication in neuroscientific studies: is it affecting your analysis? BMC Neurosci 2010 11 5 10.1186/1471-2202-11-5 20074371 PMC2817684

[B56] Peinemann A Lehner C Conrad B Siebner HR Age-related decrease in paired-pulse intracortical inhibition in the human primary motor cortex Neurosci Lett 2001 313 33 36 10.1016/S0304-3940(01)02239-X 11684333

[B57] Di Lazzaro V Oliviero A Pilato F Saturno E Dileone M Marra C Daniele A Ghirlanda S Gainotti G Tonali PA Motor cortex hyperexcitability to transcranial magnetic stimulation in Alzheimer’s disease J Neurol Neurosurg Psychiatry 2004 75 555 559 10.1136/jnnp.2003.018127 15026495 PMC1739006

[B58] Mesulam M Shaw P Mash D Weintraub S Cholinergic nucleus basalis tauopathy emerges early in the aging-MCI-AD continuum Ann Neurol 2004 55 815 828 10.1002/ana.20100 15174015

[B59] Dumas JA Newhouse PA The cholinergic hypothesis of cognitive aging revisited again: cholinergic functional compensation Pharmacol Biochem Behav 2011 99 254 261 10.1016/j.pbb.2011.02.022 21382398 PMC3114182

[B60] Lamar M Resnick SM Aging and prefrontal functions: dissociating orbitofrontal and dorsolateral abilities Neurobiol Aging 2004 25 553 558 10.1016/j.neurobiolaging.2003.06.005 15013577

[B61] Thienel R Kellermann T Schall U Voss B Reske M Halfter S Sheldrick AJ Radenbach K Habel U Shah NJ Kircher T Muscarinic antagonist effects on executive control of attention Int J Neuropsychopharmacol 2009 12 1307 1317 10.1017/S146114570999068X 19793402

[B62] Robbins TW Semple J Kumar R Truman MI Shorter J Ferraro A Fox B McKay G Matthews K Effects of scopolamine on delayed-matching-to-sample and paired associates tests of visual memory and learning in human subjects: comparison with diazepam and implications for dementia Psychopharmacol 1997 134 95 106 10.1007/s002130050430 9399372

[B63] Fredrickson A Snyder PJ Cromer J Thomas E Lewis M Maruff P The use of effect sizes to characterize the nature of cognitive change in psychopharmacological studies: an example with scopolamine Hum Psychopharmacol 2008 23 425 436 10.1002/hup.942 18421801

[B64] Hodges DB Lindner MD Hogan JB Jones KM Markus EJ Scopolamine induced deficits in a battery of rat cognitive tests: comparisons of sensitivity and specificity Behav Pharmacol 2009 20 237 251 10.1097/FBP.0b013e32832c70f5 19436198

[B65] Edginton T Rusted JM Separate and combined effects of scopolamine and nicotine on retrieval-induced forgetting Psychopharmacol 2003 170 351 357 10.1007/s00213-003-1563-2 12955293

[B66] Little JT Johnson DN Minichiello M Weingartner H Sunderland T Combined nicotinic and muscarinic blockade in elderly normal volunteers: cognitive, behavioral, and physiologic responses Neuropsychopharmacol 1998 19 60 69 10.1016/S0893-133X(98)00002-5 9608577

[B67] Mesulam M Siddique T Cohen B Cholinergic denervation in a pure multi-infarct state: observations on CADASIL Neurology 2003 60 1183 1185 10.1212/01.WNL.0000055927.22611.EB 12682331

[B68] Roman GC Cholinergic dysfunction in vascular dementia Curr Psychiatry Rep 2005 7 18 26 10.1007/s11920-005-0019-2 15717981

[B69] Easton A Fitchett AE Eacott MJ Baxter MG Medial septal cholinergic neurons are necessary for context-place memory but not episodic-like memory Hippocampus 2011 21 1021 1027 20842629 10.1002/hipo.20814

[B70] Rusted JM Trawley S Heath J Kettle G Walker H Nicotine improves memory for delayed intentions Psychopharmacology (Berl) 2005 182 355 365 10.1007/s00213-005-0109-1 16160879

[B71] Parikh V Sarter M Cholinergic mediation of attention: contributions of phasic and tonic increases in prefrontal cholinergic activity Ann N Y Acad Sci 2008 1129 225 235 10.1196/annals.1417.021 18591483

[B72] Parikh V Kozak R Martinez V Sarter M Prefrontal acetylcholine release controls cue detection on multiple timescales Neuron 2007 56 141 154 10.1016/j.neuron.2007.08.025 17920021 PMC2084212

[B73] Shinotoh H Namba H Fukushi K Nagatsuka S Tanaka N Aotsuka A Ota T Tanada S Irie T Progressive loss of cortical acetylcholinesterase activity in association with cognitive decline in Alzheimer’s disease: a positron emission tomography study Ann Neurol 2000 48 194 200 10.1002/1531-8249(200008)48:2<194::AID-ANA9>3.0.CO;2-X 10939570

[B74] Salthouse TA When does age-related cognitive decline begin? Neurobiol Aging 2009 30 507 514 10.1016/j.neurobiolaging.2008.09.023 19231028 PMC2683339

[B75] Nilsson LG Sternang O Ronnlund M Nyberg L Challenging the notion of an early-onset of cognitive decline Neurobiol Aging 2009 30 521 524 discussion 530–523 10.1016/j.neurobiolaging.2008.11.013 19285194

[B76] Di Lazzaro V Oliviero A Saturno E Dileone M Pilato F Nardone R Ranieri F Musumeci G Fiorilla T Tonali P Effects of lorazepam on short latency afferent inhibition and short latency intracortical inhibition in humans J Physiol 2005 564 661 668 10.1113/jphysiol.2004.061747 15718269 PMC1464438

[B77] Martorana A Mori F Esposito Z Kusayanagi H Monteleone F Codeca C Sancesario G Bernardi G Koch G Dopamine modulates cholinergic cortical excitability in Alzheimer’s disease patients Neuropsychopharmacol 2009 34 2323 2328 10.1038/npp.2009.60 19516251

[B78] Yu ZY Wang W Fritschy JM Witte OW Redecker C Changes in neocortical and hippocampal GABAA receptor subunit distribution during brain maturation and aging Brain Res 2006 1099 73 81 10.1016/j.brainres.2006.04.118 16781682

[B79] Di Lazzaro V Pilato F Dileone M Tonali PA Ziemann U Dissociated effects of diazepam and lorazepam on short-latency afferent inhibition J Physiol 2005 569 315 323 10.1113/jphysiol.2005.092155 16141274 PMC1464195

[B80] Dennis NA Cabeza R Craik FIM, Salthouse TA Neuroimaging of healthy cognitive aging Handbook of Aging and Cognition 2008 3 Eribaum, Mahwah, NJ 1 54

[B81] Raz N Rodrigue KM Differential aging of the brain: patterns, cognitive correlates and modifiers Neurosci Biobehav Rev 2006 30 730 748 10.1016/j.neubiorev.2006.07.001 16919333 PMC6601348

[B82] Yankner BA Lu T Loerch P The aging brain Annu Rev Pathol 2008 3 41 66 10.1146/annurev.pathmechdis.2.010506.092044 18039130

[B83] Kalpouzos G Persson J Nyberg L Local brain atrophy accounts for functional activity differences in normal aging Neurobiol Aging 2012 33 623.e1 623.e13 10.1016/j.neurobiolaging.2011.02.021 21524432

[B84] Van Petten C Plante E Davidson PS Kuo TY Bajuscak L Glisky EL Memory and executive function in older adults: relationships with temporal and prefrontal gray matter volumes and white matter hyperintensities Neuropsychologia 2004 42 1313 1335 10.1016/j.neuropsychologia.2004.02.009 15193940

[B85] Ryan L Walther K Bendlin B Lue L Walker DG Glisky E Age-related differences in white matter integrity and cognitive function are related to APOE status Neuroimage 2011 54 1565 1577 10.1016/j.neuroimage.2010.08.052 20804847 PMC2997188

